# Neighbourhood exposure to fast-food and sit-down restaurants and estimated 24-hour urinary sodium excretion: a cross-sectional analysis of urban adults from the ORISCAV-LUX 2 study

**DOI:** 10.1017/S1368980025000540

**Published:** 2025-04-21

**Authors:** Marion Tharrey, Olivier Klein, Torsten Bohn, Dmitry Bulaev, Juliette Van Beek, Laurent Malisoux, Camille Perchoux

**Affiliations:** 1 Department of Urban Development and Mobility, Luxembourg Institute of Socio-Economic Research, 11 Porte des Sciences, 4366, Esch-sur-Alzette, Luxembourg; 2 Department of Precision Health, Luxembourg Institute of Health, 1A-B Rue Thomas Edison, 1445, Strassen, Luxembourg; 3 Competence Center for Methodology and Statistics, Luxembourg Institute of Health, 1A-B Rue Thomas Edison, 1445, Strassen, Luxembourg; 4 Faculty of Humanities, Education and Social Sciences, Department of Geography and Spatial Planning, University of Luxembourg, 11 Porte Des Sciences, 4366, Esch-Sur-Alzette, Luxembourg

**Keywords:** Restaurants, Sodium, Cross-sectional, Geographic information system, Neighbourhood effect

## Abstract

**Objective:**

Increased out-of-home consumption may elevate sodium (Na) intake, but self-reported dietary assessments limit evidence. This study explored associations between neighbourhood exposure to fast-food and sit-down restaurants and estimated 24-hour urinary Na excretion.

**Design:**

A cross-sectional analysis from the ORISCAV-LUX 2 study (2016–2017). 24-hour urinary Na was estimated from a morning spot urine sample using the INTERSALT formula. Spatial access to fast-food and sit-down restaurants was derived from GIS data around participants’ addresses within 800-m and 1000-m road network buffers by summing up the inverse of the road network distance between their residential address and all restaurants within the corresponding buffer size. Multi-adjusted linear models were used to assess the association between spatial access to restaurants and estimated 24-hour urinary Na excretion.

**Setting:**

Luxembourg

**Participants:**

Urban adults age over 18 years (*n* 464).

**Results:**

Fast-food and sit-down restaurants accounted for 58·5 % of total food outlets. Mean 24-hour urinary Na excretion was 3564 mg/d for men and 2493 mg/d for women. Health-conscious eating habits moderated associations between spatial access to fast-food and sit-down restaurants and Na excretion. For participants who did not attach great importance to having a balanced diet, greater spatial access to restaurants, combining both density and accessibility, was associated with increased urinary Na excretion at 800 m (*β*_highvslow_ = 259, 95 % CI: 47, 488) and 1000 m (*β*_highvslow_ = 270, 95 % CI: 21, 520).

**Conclusions:**

Neighbourhood exposure to fast-food and sit-down restaurants influences Na intake, especially among individuals with less health-conscious eating habits, potentially exacerbating diet-related health disparities.

Poor diet quality and nutrition are major preventable risk factors for overweight and obesity, as well as for diet-related non-communicable diseases, such as cardiovascular diseases, cancer and diabetes^(1)^, which are the leading causes of death worldwide^(2)^. High intake of sodium (Na) ranks as the number one dietary risk factor for non-communicable diseases, accounting for approximately 3 million deaths and 70 million disability-adjusted life years worldwide in 2017^(1)^. Salt affects health not only by damaging target organs predominantly by raising blood pressure but also through hormonal and inflammatory pathways, as well as by influencing immune responses and the gut microbiome^(3)^. The burden of non-communicable diseases attributable to the excessive intake of Na is particular high in European countries^(1)^, where dietary habits include a high proportion of bread, bakery products, processed meats, dairy products, sauces and convenience meals, which are the leading contributors to Na intake in most populations^(4)^. Despite the recommendation of the WHO to keep personal salt intake to less than 2000 mg/d of Na (equivalent to 5 g/d of Na chloride), Na intake in the WHO European Region is substantially above those recommended levels^(5)^. In Luxembourg, the estimated median per capita intake of dietary Na exceeded recommendations in 2007–2008 and continued to rise over the subsequent decade, from 2332 to 3333 mg/d^(6)^.

In response to the shift in dietary patterns influenced by the increased supply, accessibility and marketing of energy-dense, nutrient-poor processed foods, European public policy initiatives have turned their attention to the local food environment, which encompasses ‘the collective physical, economic, policy and socio-cultural surroundings’^(7)^, in order to reduce salt intake at the population level^(8)^. Of particular concern is the rise of fast food, which encompasses a variety of quick-service establishments, generally offering pre-prepared food with minimal table service (hamburgers, pizza chains, etc.) and sit-down restaurants, which typically include establishments where customers are seated and a member of staff takes their order. Both have been reported to offer Na-rich foods, regardless of the type of establishment^(9)^. A growing body of observational studies suggests that the shift towards out-of-home consumption over recent decades has contributed to increased Na intake^(10,11)^. However, the limited number of studies, variability in methods and reliance on self-reported dietary assessments have hindered the available evidence, leading to mixed results^(10)^ and underscoring the need for more objective measurements in assessing both the food environment and Na intake. In this regard, geographic information systems have been increasingly used in epidemiological research to examine the role of ‘place’ as a contextual element of different health and diet-related risk factors^(12)^. While some studies have found links between neighbourhood availability of and proximity to fast-food outlets, increased fast-food consumption and poorer diet quality, the overall evidence remains inconsistent^(13)^. The link between exposure to sit-down restaurants and diet has been even less explored^(13)^, and to the best of our knowledge, no study has examined the exposure to fast-food and sit-down restaurants in relation to Na intake.

It has been acknowledged that heterogeneity in the assessment of environmental exposure related to the definition of a neighbourhood, its size and the type measurements (e.g. distance, density, proportion of food outlets), is a major methodological challenge that hampers the collation and interpretation of results linking the food environment and diet^(13,14)^. However, it is also likely that people respond differently to their environment, depending on personal and contextual factors, potentially mitigating the association between food environment and dietary behaviour^(15)^. For instance, education, income, culture, pro-health behaviours and physical access to healthy food options are among many factors that could affect food habits^(16)^. In that regard, men and more disadvantaged populations are more inclined towards takeaway and fast-food consumption.^(17)^ Compared with women, they could therefore exhibit a stronger association between exposure to fast-food and sit-down restaurants and Na intake. Pro-health attitudes may further influence the impact of exposure to fast-food and sit-down restaurants on eating habits^(18)^, as individuals who value healthy eating may be less inclined to dine out in order to avoid high-fat and/or high-Na foods and maintain greater control over their diet.

The purpose of this study was to examine the cross-sectional association between neighbourhood exposure to fast-food and sit-down restaurants and salt intake among adults from a nationwide population-based survey in the Grand Duchy of Luxembourg. As self-reported dietary assessments (e.g. 24-hour recall or diet records) generally yield inaccurate estimates of Na intake^(19)^, we used estimated 24-hour urinary Na excretion based on spot urine samples. We hypothesised that greater access to fast-food and sit-down restaurants would be associated with higher 24-hour urinary Na excretion. We expected associations to be stronger for men, disadvantaged populations and/or those with less pro-health behaviours.

## Methods

### Study design, data and participants

**Figure 1.**
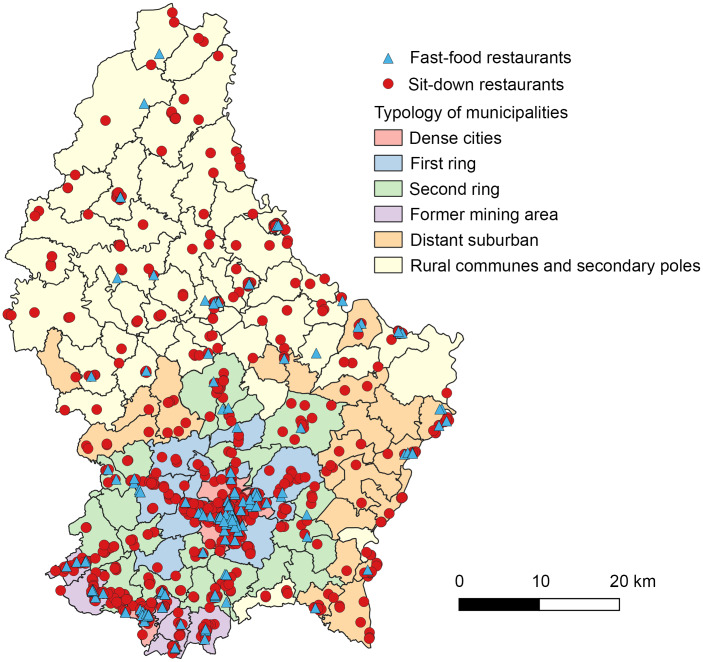
Map of fast-food outlets (*n* 213) and sit-down restaurants (*n* 1335) in Luxembourg in 2017 (STATEC, 2017).

This study uses data from the cross-sectional Observation of Cardiovascular Risk Factors in Luxembourg (ORISCAV-LUX 2) study. A nationwide population-based survey conducted from 2016 to 2017 among the adult population of the Grand Duchy of Luxembourg. Details of the study design have been previously published^(20)^. Briefly, ORISCAV-LUX 2 was a follow-up of the ORISCAV-LUX 1 study conducted in 2007–2009 in Luxembourg to monitor the cardiometabolic health of the population^(21)^. The ORISCAV-LUX 2 sample included 1558 adults aged 25–79 years recruited according to different random sampling strategies. Participants from the first national survey (ORISCAV-LUX 1) were recontacted. This first wave was a nationally representative sample of 1432 subjects aged 18–69, created by random sampling stratified on age, sex and district from the National Health Insurance Register (IGSS)^(21)^. A sample of 660 participants enrolled in the first wave agreed to take part in the second survey, for which three additional alternative sampling strategies were implemented to overcome the drop in the number of participants for the follow-up (second wave)^(20)^. All the participants filled in a self-reported questionnaire at home, followed by a nurse interview combined with anthropometric and clinical examination. In 2021, a letter was sent to the 660 participants who participated in both waves (ORISCAV-LUX 1 and 2), asking for their agreement for secondary data analyses, using their data and address coordinates to extract information regarding the environmental characteristics of their residential neighbourhood at both time points. In response to this, 633 (96·6 %) gave informed consent. From this sample, we excluded participants living in rural municipalities (*n* 122), as they were little exposed to fast-food and sit-down restaurants (Figure 1), as well as those self-reporting chronic conditions (heart failure, myocardial infarction, cerebrovascular accident or cancer) (*n* 47), resulting in a final sample of 464 participants.

### 24-hour urinary sodium excretion

As around 90 % of daily Na intake is excreted in the urine throughout the day^(22)^, the measurement of 24-hour urinary Na excretion is considered the gold standard for assessing dietary Na intake^(19,23)^. However, the collection of complete 24-hour urine Na excretion is tedious and not feasible in large epidemiological studies due to the high participant burden and cost. Therefore, spot urine samples have been identified as an appropriate alternative to measure population salt intake^(24)^. In the ORISCAV-LUX 2 survey, morning fasting spot urine samples were collected. Participants were invited to go to the Ketterthill laboratory (LKT, an accredited commercial laboratory) for sample collection. Samples were then transported to the LKT central facility to be processed and shipped daily to the Integrated BioBank of Luxembourg (IBBL, Dudelange, Luxembourg) for storage. The INTERSALT formula developed in the Western population was used to estimate total 24-hour urinary Na from a spot urine sample^(25)^, providing a more practical alternative to time-consuming complete 24-hour urine collection. This validated equation takes into account the Na, potassium (K) and creatinine concentrations of spot urine samples and individual characteristics such as age, sex, geographical region and BMI to approximate daily Na intake (see online supplementary material, Supplemental Table 1)^(25)^. As total urinary creatinine excretion remains relatively stable throughout the day and is hardly impacted by food intake, correcting for its concentration aids in considering the urinary output volume that can be variable during the day. The correction with potassium is typically carried out as an inverse relation between Na intake and potassium intake – and therefore excretion – was observed. Hence, the combined equation provides, on the population level, a good approximation of total daily Na excretion.

### Exposure to fast-food and sit-down restaurants

#### Food outlet data

Data collection and reporting were carried out in accordance with the Geo-FERN reporting framework (see online supplementary material, Supplemental Table 2). The list of food outlets was obtained from the Luxembourg business directory data coordinated by the National Institute of Statistics and Economic Studies of the Grand Duchy of Luxembourg (STATEC 2017). The directory contains each entity’s name, address and economic activity code according to the NACE Rev. 2 classification^(26)^ of all registered businesses in the country. Restaurants were selected using the NACE codes 56·1 (restaurants and mobile food service activities). As fast-food and sit-down restaurants are not classified under a specific NACE code, we referred to the Atlas du Luxembourg, which provides a typology of restaurants in Luxembourg (including the name and type of cuisine), allowing us to differentiate fast-food and sit-down restaurants. The *Atlas du Luxembourg* (2009) was used as a reference, and an update was made for the second wave (2017) based on the type of restaurant/cuisine and information collected on the web. Fast-food restaurants were classified on the basis of (i) their classification in the 2009 Atlas of Luxembourg, (ii) the name of the restaurant/chain (e.g. McDonald’s, Burger King, KFC, etc.), (iii) the impossibility for the customers to order food while seated at their table and (iv) their designation as ‘fast-food restaurants’ on food delivery websites, when available. We validated our classification by cross-referencing it with Open Street Map (2017) and amenities datasets provided by the Spatial Development Observatory from the Ministry of Housing and Spatial Planning of the Grand Duchy of Luxembourg.

#### Exposure measurements

To account for both density and proximity, we computed the spatial access to fast-food and sit-down restaurants, which we previously found to be a relevant metric to capture the healthiness of food environments in Luxembourg^(27)^. For each participant, spatial access to fast-food and sit-down restaurants was calculated by summing the inverse of the road network distance in meters (m) between the residential address and all restaurants within a predefined road network buffer. Higher values indicate that participants live closer to a larger number of restaurants^(28)^. The further away the fast-food and sit-down restaurants are from participants’ residences, the less accessible these restaurants become. The variable is defined as follows:

Spatial access to fast-food and sit-down restaurants (m^–1^) = ∑(1/street network distance fast-food and sit-down restaurants in the road network buffer)

Spatial access measurements were calculated at 800 and 1000 m road-network buffer size around each participant’s residential address. The reason for using these buffer sizes was twofold: First, 800 and 1000 m buffer sizes are a commonly used metric for defining residential neighbourhood food environments (equivalent to a 15–20 min walk).^(29)^ Second, smaller buffer sizes were deemed too small as they could lead to an excessive number of participants without exposure to fast-food and sit-down restaurants, while larger buffer sizes are more likely to be travelled by car and therefore are not a realistic representation of the proximity within neighbourhoods.

Participants’ residential addresses and food outlet addresses were geolocated with the georeferenced addresses database (BD Addresses), provided by the Administration of Cadastre and Topography (ACT), using ArcGIS (Version 9.3.1; ESRI, Redlands, CA, USA, 2010). The road network was obtained from the BD-L-TC topo-cartographic database (2015 version) provided by the Administration of Cadaster and Topography (Luxembourg City, Luxembourg).

#### Covariates

To guide the identification of potential confounders, we constructed a directed acyclic graph as shown in the online supplementary material, Supplemental Figure 1. Directed acyclic graphs are visual representations that depict a priori causal assumptions regarding the relationships between variables^(30)^.

Individual-level covariates were obtained from the self-administered questionnaire and include sex, age, country of birth (Luxembourg, European country, or non-European country), resource perception (difficult, easy or refuse to answer), educational level (no diploma, secondary education or higher diploma), work status (employed, not employed, stay-at-home parent, disabled/retired), marital status (married/living with partner, single/never married or divorced/widowed), presence of a child in the household (yes or no), attitude towards healthy eating (great or enough/little importance) and attitude towards weight management (great or enough/little importance). For the latter two variables, participants were asked about the importance they placed on having a balanced meals and maintaining a normal weight for good health. The response options included ‘great importance’, ‘enough importance’, ‘little importance’ and ‘no importance’ (which no participant selected). ‘Enough importance’ and ‘little importance’ were combined to differentiate those who attached great importance to having a balanced diet from those who did not.

Neighbourhood-level covariates include neighbourhood socio-economic status (SES) and the healthiness of the retail food environment. Patterns of neighbourhood SES were derived by principal component analysis, based on six variables: percentage of unemployment, percentage of blue-collar workers, monthly gross total wage (in euros), percentage of domestic community receiving the guaranteed minimum income supplementary allowance, percentage of the domestic community receiving cost-of-living allowance and average housing sales prices (in euros per m^2^). Considering eigenvalues > 1 and a breakpoint in the Scree test, two factors explaining > 80 % of total variance were retained. The first factor represents relatively deprived neighbourhoods with high loadings on receiving minimum income and cost-of-living allowances and low loadings on monthly total wage. The second factor describes higher neighbourhood SES with high loadings on average housing sales prices and blue-collar workers and low loading on unemployment (see online supplementary material, Supplemental Table 3). Details regarding the extraction of the factors have been described elsewhere^(27)^. The healthiness of the retail food environment was assessed in 800 m and 1000 m road network buffers around the participant’s address by the modified Retail Food Environment Index^(31)^, a commonly used indicator to assess the relative density of healthy food retailers. The modified Retail Food Environment Index (range: 0–100) was calculated as the ratio between the number of healthy food retailers – that is supermarkets and specialised shops selling fresh fruit and vegetables (greengrocers and open markets) – and the total number of food retailers within each participant’s neighbourhood^(31)^, including healthy food retailers plus small grocers, convenience stores (which are located at petrol stations in Luxembourg), bakeries, butchers, fishmongers and restaurants. Online supplementary material, Supplemental Table 4 shows details of the methods and sources. The final modified Retail Food Environment Index was categorised into tertiles.

### Statistical analysis

The socio-demographic characteristics of the population were compared between men and women via a two-sample *t* test for continuous variables (following assessment of normality and homogeneity assumptions) and Fisher’s exact tests for categorical variables. To handle missing values, multiple imputation by chained equations was employed using fully conditional specification method^(32)^. All the variables used in this study were incorporated in the imputation model^(33)^. The number of imputed datasets generated was set equal to the percentage of incomplete cases^(34)^. Imputation was performed using the *mice* R package. A total of twenty imputed datasets were created and used in subsequent analyses.

We used general linear models (PROC GLM procedure in SAS, SAS Institute, Inc., Cary, North Carolina) to assess the association between spatial access to fast-food and sit-down restaurants and estimated 24-hour urinary Na excretion. Spatial access to fast-food and sit-down restaurants was categorised into low, intermediate or high, based on tertiles. A significant interaction between age and sex (F-test, *P* < 0·0001) was observed in all imputed datasets and was therefore added to the models. First, two models with increasing levels of adjustment were tested. Model 1 was adjusted on individual-level covariates, and Model 2 was further adjusted on neighbourhood-level covariates. Estimates (*β*) and 95 % CI from the twenty imputed datasets were summarised using PROC MIANALYZE in SAS. Then, effect modifications by sex, age, individual SES and neighbourhood SES, as well as pro-health behaviours were investigated by adding multiplicative interaction terms with spatial access to fast-food and sit-down restaurants. We only retained statistically significant interaction terms according to a type III test (F-test, *P*-value < 0·05), comparing the models with and without interactions. The assumption of normality and homoscedasticity of the residuals was checked visually for each set of imputed data. Measurements of skewness (±1) and kurtosis (±2) also showed no sign of deviation from normality.

Multiple imputations were performed in R version 4.3.1. All statistical analyses were performed using SAS version 9.4 (SAS Institute), and a two-tailed *P*-value < 0·05 was considered as statistically significant.

### Sensitivity analyses

To disentangle the specific role of each type of restaurant on urinary Na levels, we ran a model considering solely sit-down restaurants. Given the high number of participants with no exposure to fast-food restaurants (see online supplementary material, Supplemental Table 5), it was not possible to run a model including only fast-food outlets. We therefore ran sensitivity analyses on the association between the presence of fast-food restaurants, the count of sit-down restaurants and the count of fast-food and sit-down restaurants and estimated 24-hour urinary Na excretion. Presence of fast-food restaurants was dichotomised into yes *v*. no, while the count of sit-down restaurants and the count of fast-food and sit-down restaurants were categorised into low, intermediate or high, based on tertiles. We also tested sensitivity to the imputation by running the analyses on the non-imputed data (complete case analysis).

## Results

### Study population

Mean (sd) urinary excretion of Na was 3564 (744) mg/d for men and 2493 (862) mg/d for women. Men and women had similar socio-demographic characteristics except for work status, as women were more likely to be unemployed or a stay-at-home parent (Table 1).

**Table 1.** Socio-demographic characteristics of the study population by sex, *n* 464 adults from ORISCAV-LUX 2 study

	Men (*n* 229)		Women (*n* 235)		*P*-value^*^
Estimated 24-hour Urinary Na excretion, mg/d	3564 (744)		2493 (862)		< 0·0001
	Mean	sd	Mean	sd	
Mean (sd) age, years	51·2	11·8	53·2	11·6	0·748
	*n* (%)		*n* (%)		
Resource perception, *n* (%)					0·895
Difficult	36	15·7	38	16·2	
Easy	153	66·8	155	66·0	
Refuse to answer	37	16·2	42	17·8	
N/A	3	1·3	0	0	
Work status, *n* (%)					< 0·0001
Employed	162	70·8	135	57·5	
Not employed/stay-at-home parent	6	2·6	44	18·7	
Disabled or retired	58	25·3	55	23·4	
NA	3	1·3	1	0·4	
Education level, *n* (%)					0·589
No diploma	34	14·8	38	16·2	
High school or vocational diploma	96	41·9	107	45·5	
Higher diploma	97	42·4	89	37·9	
N/A	2	0·9	1	0·4	
Marital status, *n* (%)					0·150
Married/living with partner	177	77·3	169	71·9	
Single/never married	24	10·5	22	9·4	
Divorced/widowed	27	11·8	43	18·3	
N/A	1	0·4	1	0·4	
Country of birth, *n* (%)					0·782
Luxembourg	136	59·4	140	59·6	
European country	78	34·1	84	35·7	
Non-European country	15	6·5	11	4·7	
Presence of a child in the household, *n* (%)					0·782
Yes	138	60·3	162	68·9	
No	89	38·8	73	31·1	
N/A	2	0·9	0	0	
Great importance attached to eating balanced meals for good health, *n* (%)					0·351
Yes	111	48·5	127	54·0	
No	113	49·3	108	46·0	
N/A	5	2·2	0	0	
Great importance attached to maintaining normal weight for good health, *n* (%)					0·640
Yes	99	43·2	109	46·4	
No	126	55·0	126	53·6	
N/A	4	1·8	0	0	

* *P*-value from a two-sample *t* test (continuous variables, equal variances), or, Welch’s *t* test (continuous variables, unequal variances) or, Fisher’s exact test of significance of association (categorical variables).

### Exposure to fast-food and sit-down restaurants

For 2017, we counted 213 fast-food and 1335 sit-down restaurants, primarily located in urbanised municipalities (Figure 1), accounting for 58·5 % of the total food outlets in the country (data not shown). The median number of fast-food and sit-down restaurants combined within the participants’ residential neighbourhood were 3 at 800 m and 4 at 1000 m, with a median shortest distance of 495 m (see online supplementary material, Supplemental Table 5). Spatial access to fast-food and sit-down restaurants was greater in dense cities (Luxembourg and Esch-sur-Alzette), followed by former mining areas in the southern region of the country (see online supplementary material, Supplemental Table 6). The median (IQR) of spatial access to fast-food and sit-down restaurants, as well as tertiles are shown in online supplementary material, Supplemental Table 5.

### Spatial access to fast-food and sit-down restaurants and estimated 24-hour urinary Na excretion

**Figure 2.**
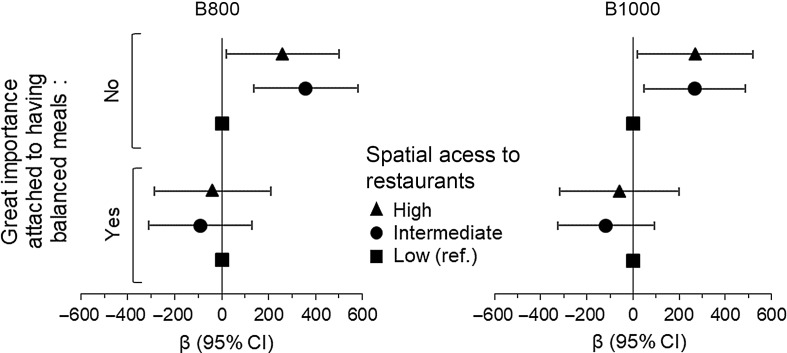
Estimates (*β*) and 95 % CI for associations of spatial access to restaurants and 24-hour urinary Na excretion (mg/d), at 800 m and 1000 m, according to health-conscious eating habits. Fully adjusted model (Model 2).

No significant associations were found between spatial access to fast-food and sit-down restaurants and 24-hour urinary Na excretion in the total population (Table 2). Analyses revealed a significant interaction term between attitude towards healthy eating and spatial access to fast-food and sit-down restaurants at 800 m (F-test, *P*-value < 0·05: 95 % of imputed datasets) and 1000 m (F-test, *P*-value < 0·05: 90 % of imputed datasets). For participants who did not attach great importance to having balanced meals only, a greater exposure to fast-food and sit-down restaurants was associated with higher 24-hour urinary Na excretion at 800 m (*β*_intermediate_ = 358, 95 % CI: 136, 580; *β*_high_ = 259, 95 % CI: 20, 499) and 1000 m (*β*_intermediate_ = 268, 95 % CI: 47, 488; *β*_high_ = 270, 95 % CI: 21, 520), as shown in Figure 2. Detailed coefficients are presented in online supplementary material, Supplemental Table 7. There was no significant association between spatial access to fast-food and sit-down restaurants and 24-hour urinary Na excretion for participants who reported more health-conscious eating habits. Sex, individual SES and both factors of neighbourhood SES did not moderate the associations between spatial access to fast-food and sit-down restaurants and estimated 24-hour urinary Na excretion (data not shown).

**Table 2.** Estimates (*β*) and 95 % CI for associations of spatial access to fast-food and sit-down restaurants, and 24-hour urinary Na excretion (mg/d), by different road network buffer sizes

	800 m			1000 m		
Tertiles of spatial access	*β*	95 % CI	*P*-value	*β*	95 % CI	*P*-value
Model 1^*^						
Low	ref.		–	ref.		–
Intermediate	135·5	–19·8, 290·9	0·087	79·2	–69·7, 228·1	0·297
High	105·2	–42·7, 253	0·163	104	–44·4, 252·4	0·169
Model 2^†^						
Low	ref.		–	ref.		–
Intermediate	133·2	–22·5, 288·8	0·094	77·9	–76·2, 232	0·322
High	109·8	–78·4, 298·1	0·253	109·7	–92·8, 312·2	0·288

* Model 1 was adjusted for sex, age, country of birth (Luxembourg, European country or non-European country), resource perception (difficult, easy or refuse to answer), educational level (no diploma, secondary education or higher diploma), work status (employed, not employed, stay-at-home parent or disabled/ retired), marital status (married/living with partner, single/never married or divorced/widowed), presence of a child in the household (yes or no), great importance attached to eating balanced meals for good health (yes or no) and great importance attached to maintaining normal weight for good health (yes or no).

† Model 2 = Model 1 + tertiles of modified Retail Food Environment Index, as well as two scores of neighbourhood SES derived from principal component analysis.

The results were consistent when running the analyses on the non-imputed dataset (see online supplementary material, Supplemental Table 8 and Supplemental Figure 2). Sensitivity analyses performed only for sit-down restaurants showed similar results (see online supplementary material, Supplemental Table 9). Additionally, a significant interaction term was observed between neighbourhood SES (Factor 2) and spatial access to sit-down restaurants at 800 m and 1000 m (F-test *P*-value < 0·05: > 75 % imputed datasets). After categorizing Factor 2 into tertiles to facilitate visual representation of the interaction, we found that a greater exposure to sit-down restaurants was associated with a greater 24-hour urinary Na excretion at 800 m (*β*_intermediate_ = 384, 95 % CI: 101, 667; *β*_high_ = 302, 95 % CI: 22, 583) and 1000 m (*β*_intermediate_ = 334, 95 % CI: 48, 621; *β*_high_ = 286, 95 % CI: 0·7, 571) for participants in the upper tertile of Factor 2 but not the lower tertile (see online supplementary material, Supplemental Figure 3). Sensitivity analyses performed on the presence of fast-food restaurants only, counts of sit-down restaurants only and counts of fast-food and sit-down restaurants showed no associations except for the presence of fast-food restaurants only (see online supplementary material, Supplemental Table 10). The presence of fast-food restaurants in an 800 m road network buffer was inversely correlated with 24-h urinary Na excretion (*P*-value = 0·036). This finding may be explained by the fact that the (road) distance to the outlet and their density in the buffer were not considered as a covariate, as compared to spatial access. When the spatial access to fast-food and sit-down restaurants is used instead, the observed trend inverts – the association between spatial access and 24-h urinary Na excretion becomes positive.

## Discussion

To our knowledge, this is the first study to investigate the association between neighbourhood exposure to fast-food and sit-down restaurants and urinary Na excretion. Using data for urban adults from a nationwide population-based survey and spot urine samples, we found that the mean daily Na intake as estimated by the INTERSALT formula exceeded the WHO recommendation of 2000 mg/d of Na intake for both men and women. Greater neighbourhood exposure to fast-food and sit-down restaurants was associated with higher estimated 24-hour urinary Na excretion, though only among participants who did not report attaching great importance to having balanced meals. Sex, individual SES and neighbourhood SES did not moderate the associations between spatial access to fast-food and sit-down restaurants and estimated 24-hour urinary Na excretion.

To date, only a limited number of studies have explored the effects of objective exposure to the food environment on Na intake, yielding inconclusive findings. In the UK, analyses of large-scale objective commercial purchasing data revealed no significant association between density and distance to restaurants and takeaway outlets and purchases of foods and drinks high in fat, salt and sugar; however, Na intake was not studied in isolation^(35)^. In the United States, no association was found between the modified Retail Food Environment Index (measuring the relative density of healthy and less healthy food retailers) and Na intake, although it is notable that sit-down restaurants were not encompassed by the analysis^(36)^. In Japan, a study conducted on female dietetic students showed no association between various metrics of neighbourhood food store availability and urinary Na excretion^(37)^. Our study contributes to this strand of literature by investigating the specific role of neighbourhood exposure to restaurants in urban settings. We found no clear evidence of any association between exposure to fast-food and sit-down restaurants and urinary Na excretion in the total sample. Sensitivity analyses on presence and spatial access to sit-down restaurants only yielded similar results, while we observed an unexpected negative association between the presence of fast-food restaurant in an 800 m buffer from the residence and urinary Na excretion (association not sustained at 1000 m). These findings, in conjunction, may suggest that the presence (*v*. absence) of restaurant is not fully capturing the mechanisms of accessibility, yielding contradictory findings. The sole presence of fast-food restaurants in the neighbourhood is not necessarily linked with higher Na excretion levels, and the distance to those restaurants needs to be considered, since it demonstrates to strongly alter the effect size and direction.

Importantly, we discovered that greater exposure to fast-food outlets and sit-down restaurants was associated with higher urinary Na excretion only among participants who did not attach great importance to eating balanced meals, providing a novel insight into the moderating role of health-conscious eating habits in the relationship between the food environment and Na intake. It is well-know that out-of-home food consumption can be affected by various individual experiences, behaviours and attitude^(17)^. In particular, nutrition-related health consciousness has been recognised as an important factor shaping behaviours in terms of restaurant selection^(38,39)^ food choice^(40)^ and healthy eating^(41,42)^. Our findings suggest that being in the immediate environment of fast-food and sit-down restaurants could encourage poorer dietary habits, particularly among those who are already at greater risk of having less healthy diets, raising concerns about the potential role of the local food environment in increasing diet-related health disparities.

Sex, age and individual SES did not moderate the association between exposure to restaurants and estimated Na excretion, yet they are known as important factors affecting patterns of eating out-of-home food consumption^(17,43)^. Existing research suggests that men, younger adults and socio-economically disadvantaged populations are more inclined to opt for fast-food outlets, while women with higher socio-economic characteristics may be more likely to frequent sit-down restaurants^(17,43)^. It is likely that combining fast-food outlets and sit-down restaurants may have attenuated the moderating effect of socio-economic factors on the overall association. In line with this hypothesis, we found that spatial access to only sit-down restaurants was associated with higher Na excretion for participants living in higher SES neighbourhoods, reinforcing the idea that individuals with higher socio-economic characteristics may be more likely to frequent this type of restaurants. An expanding body of literature indicates that there are neighbourhood disparities in the food environment, with individuals living in more deprived neighbourhoods having an increased likelihood of facing the double burden of individual deprivation and greater exposure to less healthy environments^(15)^. Conversely, our study raises questions about the potential effect of the increase in the number of sit-down restaurants in the downtown areas of dense cities (generally affluent neighbourhoods) on the salt consumption of local residents. Interestingly, the importance attached to maintaining a normal weight for good health did not moderate the association between exposure to fast-food and sit-down restaurants and Na intake. This finding is consistent with the results of a previous study, suggesting that healthy food choice motives and concern about consuming too many calories are better predictors than weight control in the formation of healthy eating attitudes^(44)^.

Na levels in major fast-foods and chain restaurants are high but with great variability between companies and countries^(45–47)^. Yet obtaining data for private restaurants is challenging, as recipes vary widely between establishments and chefs. The persistence of the associations, even when analyses were restricted to sit-down restaurants, nevertheless raises concerns regarding the Na content of restaurant meals. Member states of the WHO European Region have demonstrated significant leadership and progress in efforts to reduce population-level salt consumption, with about half of the countries reporting that they have fully implemented national policies on salt reduction^(8)^. However, these policies have not been implemented in Luxembourg. As recent data from the National Institute of Statistics of Luxembourg reveals a 56 % increase between 2019 and 2022 in average household spending on eating out, and home delivery in particular^(48)^, our results call for greater policy attention to develop national salt reduction strategies targeting the food service sector.

The strengths of this study include a nationwide sample, the use of objective measurements for both exposure and outcome variables and adjustment for a large set of potential confounders. In particular, the INTERSALT formula is widely used in population-based studies for its simplicity and reliance on spot urine samples, which do not require strict participant adherence, compared with 24-hour collections. It also adjusts for urine dilution using both potassium and creatinine concentrations, improving Na intake estimation accuracy. This study also has some limitations. First, the cross-sectional design limits the ability to establish causality. Second, the drop in the number of participants between the two waves may limit the generalisability of our findings to the general population. Nevertheless, we previously found no differences in mean age and sex proportion between the population of the ORISCAV-LUX 1 study and those who also took part in ORISCAV-LUX 2^(27)^. This suggests that national representativeness for these variables was retained, even though participants in the second wave were slightly better educated^(27)^. Third, the INTERSALT formula relies on spot urine samples collected at a single time point, and this may not capture day-to-day variability in Na intake with full accuracy. The time of the day, hydration status, food intake and other factors can introduce measurement error and affect the reliability of Na intake estimates^(49)^. Fasting morning urine samples (probably not the first morning urine, as participants had to travel to the closest laboratory) were collected, thus limiting the risk of measurement error in Na excretion. However, Mann *et al.* found that the Na/creatinine ratio of a late afternoon/early evening urine sample obtained near the midpoint of a 24-hour collection strongly correlated with the actual 24-hour Na excretion, and therefore, morning samples may be more likely to underestimate 24-hour Na excretion^(49)^. Fourth, spatial access measurements did not allow for the assessment of which aspect of exposure (density or proximity) is more relevant in the association with the outcome. Although evidence indicates that availability (i.e. density) measurements may produce larger and more significant effect sizes than accessibility (i.e. proximity) measurements, both provide distinct and complementary perspectives on spatial exposure and therefore should be studied conjointly^(13)^. Last, our analysis was limited to the residential environment, thus disregarding individuals’ exposure to restaurants during daily mobility. Notably, research has shown a significant correlation between exposure to takeaway food outlets in the work neighbourhood, takeaway food consumption and BMI^(50)^. How restaurant environment influence Na intake should be further investigated in future research using activity space-based measurements.

### Conclusion

To the best of our knowledge, this study is the first to examine the association between neighbourhood exposure to fast-food and sit-down restaurants and Na excretion. Using a nationwide sample of urban adults, we found that greater spatial access to fast-food and sit-down restaurants was associated with higher estimated 24-hour urinary Na excretion among participants who did not attach great importance to having a balanced diet. These findings highlight the role of health-conscious behaviours in moderating the association between the food environment and Na intake. They suggest that pro-health attitudes may serve as a preventive factor for people with high levels of neighbourhood exposure to fast-food and sit-down restaurants. Our results underscore the need for national salt reduction strategies targeting the food service sector.

## Supporting information

Tharrey et al. supplementary material Tharrey et al. supplementary material

## Supplementary material

For supplementary material accompanying this paper visit https://doi.org/10.1017/S1368980025000540
