# Supplementary material for: Neighbourhood exposure to fast-food and sit-down restaurants and estimated 24-hour urinary sodium excretion: a cross-sectional analysis of urban adults from the ORISCAV-LUX 2 study
Source: Public Health Nutr. 2025 Apr 21;28(1):e94. doi: 10.1017/S1368980025000540 (PMC12171902; doi:10.1017/S1368980025000540)
Supplement: Tharrey et al. supplementary material [file S1368980025000540sup001.docx]

**ADIITIONAL FILES**

**TABLES**

[**Supplementary Table 1. The INTERSALT formula** 2](#_Toc195086743)

[**Supplementary Table 2. Geo-FERN reporting framework** 3](#_Toc195086744)

[**Supplementary Table 3. Factor pattern, eigenvalues, and factor loadings of the Principal Component Analysis (PCA) on neighborhood deprivation** 6](#_Toc195086745)

[**Supplementary Table 4. Methods and sources used to extract the food outlets at the national-level** 8](#_Toc195086746)

[**Supplementary Table 5. Descriptive statistics of exposure to fast-food and sit-down restaurants of the study population, n=464 adults from ORISCAV-LUX 2 study** 9](#_Toc195086747)

[**Supplementary Table 6. Descriptive statistics of exposure to fast-food and sit-down restaurants of the study population by level of urbanicity, n=464 adults from ORISCAV-LUX II study** 10](#_Toc195086748)

[**Supplementary Table 7. Estimates (β) and 95% confidence intervals (CI) for associations of spatial access to fast-food and sit-down restaurants and 24-h urinary NA excretion (mg/d)** 11](#_Toc195086749)

[**Supplementary Table 8. Estimates (β) and 95% confidence intervals (CI) for associations of spatial access to fast-food and sit-down restaurants and 24-h urinary Na excretion (mg/d), by different road network buffer sizes in the non-imputed dataset** 13](#_Toc195086750)

[**Supplementary Table 9. Estimates (β) and 95% confidence intervals (CI) for associations of spatial access to sit-down restaurants and 24-h urinary Na excretion (mg/d), by different road network buffer sizes** 14](#_Toc195086751)

[**Supplementary Table 10. Estimates (β) and 95% confidence intervals (CI) for associations of counts of fast-food, sit-down restaurants, and fast-food and sit-down restaurants and 24-h urinary Na excretion (mg/d), by different road network buffer sizes in the non-imputed dataset** 15](#_Toc195086752)

[**Supplementary Figure 1. DAG Directed Acyclic Graph (DAG) representing the causal pathways between exposure to fast-food and sit-down restaurants, and 24-hour urinary Na excretion** 18](#_Toc195086753)

[**Supplementary Figure 2. Estimates (β) and 95% confidence intervals (CI) for associations of spatial access to restaurants and 24-h urinary Na excretion (mg/d), at 800 and 1000 m, according to health-conscious eating habits in the non-imputed dataset.** 19](#_Toc195086754)

[**Supplementary Figure 3. Estimates (β) and 95% confidence intervals (CI) for associations of spatial access to sit-down restaurants and 24-h urinary Na excretion (mg/d), at 800 and 1000 m, according to health-conscious eating habits and neighborhood SES (Factor 2).** 20](#_Toc195086755)

**Supplementary Table 1. The INTERSALT formula**^(25)^

| **Sex of participants** | **Formula to estimate 24-hour urinary Na excretion (mg/d)^a^** |
| --- | --- |
| Men | 23 x [25.46 + (0.46 × spot urinary Na (mmol/L)) – (2.75 × spot urinary creatinine (mmol/L)) – (0.13 × spot urinary K (mmol/L)) + (4.10 × BMI (kg/m^2^)) + (0.26 × age (years))] |
| Women | 23 x [5.7 + (0.34 × spot urinary Na (mmol/L)) – (2.16 × spot urinary creatinine (mmol/L)) – (0.09 × spot urinary K (mmol/L)) + (2.39 × BMI (kg/m^2^)) + (2.35 × age (years)) – (0.03 × age^2^ (years))] |
| Na: sodium  ^a^ The molecular weight of Na is 23.0 g/mol. | |

# **Supplementary Table 2. Geo-FERN reporting framework**

| **Geo-FERN (Geographic Information System Food Environment Reporting) Checklist** | | |
| --- | --- | --- |
| **INSTRUCTIONS** | | |
| For each reporting item, insert a tick or cross in the shaded box to indicate whether the item has been reported, or insert ‘N/A’ if not applicable. Shading indicates whether items are essential or desirable. Reporting items can be included in supplementary materials if word limits are tight and if allowed by the publisher. | | |
| 1. **FOOD OUTLET DATA** | **Essential** | **Desirable** |
| Name of the data creator (e.g. ‘Yellow Pages’, ‘Dunn & Bradstreet’ etc.). | X |  |
| Collection and/or publication year of the data (include both if known). | X |  |
| Title of the dataset. | X |  |
| Digital identifier of the dataset (e.g. a web address or DOI). |  | X |
| Publisher of the dataset. |  | X |
| Scope of the dataset (i.e. the geographic coverage of the dataset e.g. ‘national’ or ‘regional’ and the range of businesses included in the dataset, including any notable exclusions). | X |  |
| Identification of the data fields used in analyses. | X |  |
| Original purpose of the data (e.g. food hygiene regulation enforcement or commercial business data). | X |  |
| Methods used by the data creator to collect the data/compile the dataset (e.g. audits conducted by data creator). |  | X |
| Prevalence of missing data (e.g. number of entries with incomplete address information). |  | X |
| Methods for handling missing data (e.g. case-wise deletion, or use of secondary sources to impute missing data). | X |  |
| Information on the accuracy of the data e.g. via reference to one or more validation studies or acknowledgement that data accuracy is unknown. |  | X |
| 1. **EXTRACTING FOOD OUTLETS** | **Essential** | **Desirable** |
| Description of methods used to extract food outlets of interest from dataset (e.g. search for specific proprietary classifications or store names). | X |  |
| If outlets were extracted using search terms (e.g. proprietary classifications or store names):   - An exhaustive list of search terms (where proprietary classifications are used, it should be made explicitly clear that the classifications listed are those of the data provider). | X |  |
| If outlets were extracted based on proprietary classifications:   - A copy of the proprietary classification scheme, optionally including exemplary outlets falling within each classification; OR, - A discussion of any notable categories excluded from analyses (e.g. pubs, pharmacies, mobile food vendors etc.). |  | X |
| 1. **DEFINING FOOD OUTLET CONSTRUCTS** | **Essential** | **Desirable** |
| Construct name(s) (e.g. ‘supermarkets’, ‘healthy outlets’, ‘convenience stores’ etc.). | X |  |
| Description of the methods used to group outlets into constructs, including at least one of:   - An *exhaustive* list of any list-based criteria used to define each construct. This could include e.g. proprietary classifications making up each construct, or a list of store names making up each construct. Where proprietary classifications are used, it should be made explicitly clear that the classifications listed are those of the data provider. - Any objective criteria e.g. floor space, number of tills etc. used to define constructs. - Citation of any previously published categorisation schemes that have been applied to the data and description of the methods used to apply the scheme. - Description of any other methods used (note methods based on subjective criteria are discouraged). | X |  |
| Examples of outlets falling within each construct such that the scope of each construct can be more readily interpreted. For example, if the construct ‘fast food outlet’ includes ‘traditional’ burger and fried chicken outlets, and also coffee shops and sandwich shops then well-known chains falling within each such sub-type could be listed. |  | X |
| Identification of any additional data sources used to group outlets into constructs e.g. use of Google Street View, business directories etc. | X |  |
| Description of how any additional data sources were linked to the food outlet data (e.g. by matching store names and/or addresses). |  | X |
| Where proprietary classifications are used to define constructs, a copy of the entire proprietary classification scheme. |  | X |
| 1. **GEOCODING METHODS** | **Essential** | **Desirable** |
| Acknowledgement of whether any data has been geocoded. | X |  |
| The address model used (e.g. areal unit, street segment, land parcel, address point). | X |  |
| The match rate achieved. | X |  |
| The environmental context, including details on how this was defined e.g. the study area was urban/rural, defined based on population density. | X |  |
| Geocoding software used, including the version number. | X |  |
| The source of geocoding reference data (e.g. street line segment data), including publication date. | X |  |
| 1. **ACCESS METRICS** | **Essential** | **Desirable** |
| Definition of the conceptual environment being measured e.g. home, school, work etc. | X |  |
| **Intensity Metrics** | | |
| If areal zoning system used:   - The type of areal zoning system (e.g. government districts, census tracts etc.) - The source of boundary data, including the publication date or other version identifier. | X |  |
| If buffer zoning system used:   - The buffer size. - The type of distance measure (e.g. Euclidian or network). | X |  |
| The units of the intensity metric(s) (e.g. count per unit area, as measured in meters) or formula indicating how they were calculated. | X |  |
| If network data was used (i.e. to calculate network distances):   - The source and publication date of network data. - The types of road/path included. | X |  |
| Rationale for the choice of zone type (e.g. areal vs buffer) and/or size as applicable. |  |  |
| **Proximity Metrics** | | |
| The type of distance measure (Euclidian vs network). | X |  |
| If network data was used (i.e. to calculate network distances):   - The source and publication date of network data. - The types of road/path included. | X |  |
| **Gravity Metrics** | | |
| The zone radius. | N/A |  |
| The decay coefficient. | N/A |  |
| 1. **UNKNOWN DETAILS** | **Essential** | **Desirable** |
| Any items noted as essential, but that are unknown should be highlighted as a limitation. |  |  |

Wilkins EL, Morris MA, Radley D, Griffiths C. Using Geographic Information Systems to measure retail food environments: Discussion of methodological considerations and a proposed reporting checklist (Geo-FERN). Health Place. 2017;44:110–7.

# **Supplementary Table 3. Factor pattern, eigenvalues, and factor loadings of the Principal Component Analysis (PCA) on neighborhood deprivation**

**A) At 800-m**

| **Eigenvalues of the Correlation Matrix** | | | | |
| --- | --- | --- | --- | --- |
|  | | | | |
|  | **Eigenvalue** | **Difference** | **Proportion** | **Cumulative** |
| **1** | 3.484 | 2.011 | 0.581 | 0.581 |
| **2** | 1.473 | 0.730 | 0.246 | 0.826 |
| **3** | 0.743 | 0.577 | 0.124 | 0.950 |
| **4** | 0.166 | 0.092 | 0.028 | 0.978 |
| **5** | 0.074 | 0.013 | 0.012 | 0.990 |
| **6** | 0.061 |  | 0.010 | 1.000 |

| **Rotated Factor Pattern** | | |
| --- | --- | --- |
|  | **Factor1** | **Factor2** |
| % of domestic community receiving cost-of-living allowance | 0.960 | -0.169 |
| % of domestic community receiving the guaranteed minimum income supplementary allowance | 0.937 | -0.005 |
| monthly gross total wage | -0.750 | 0.465 |
| % of blue-collar workers | -0.399 | 0.886 |
| housing price (average sales prices in euros per m²) | -0.209 | 0.833 |
| % unemployed | -0.051 | -0.816 |

**B) At 1000-m**

| **Eigenvalues of the Correlation Matrix** | | | | |
| --- | --- | --- | --- | --- |
|  | | | | |
|  | **Eigenvalue** | **Difference** | **Proportion** | **Cumulative** |
| **1** | 3.531 | 2.036 | 0.588 | 0.588 |
| **2** | 1.494 | 0.793 | 0.249 | 0.838 |
| **3** | 0.702 | 0.547 | 0.117 | 0.954 |
| **4** | 0.155 | 0.086 | 0.026 | 0.980 |
| **5** | 0.069 | 0.018 | 0.011 | 0.992 |
| **6** | 0.050 |  | 0.008 | 1.000 |

| **Rotated Factor Pattern** | | |
| --- | --- | --- |
|  | **Factor1** | **Factor2** |
| % of domestic community receiving cost-of-living allowance | 0.963 | -0.174 |
| % of domestic community receiving the guaranteed minimum income supplementary allowance | 0.947 | -0.006 |
| monthly gross total wage | -0.740 | 0.490 |
| % of blue-collar workers | -0.389 | 0.893 |
| housing price (average sales prices in euros per m²) | -0.194 | 0.851 |
| % unemployed | -0.038 | -0.820 |

# **Supplementary Table 4. Methods and sources used to extract the food outlets at the national-level**

The list of food outlets at the national level was sourced from the Luxembourg business directory data (2017), coordinated by the National Institute of Statistics and Economic Studies of the Grand Duchy of Luxembourg (STATEC), and geocoded with the Luxembourg national database of geo-referenced addresses from the Administration of Cadaster and Topography of Luxembourg. This registry contains the names, addresses, and statistical classifications of economic activities in the European Community (NACE code Rev. 2)^(1)^ for all the registered businesses in Luxembourg. Six types of food outlets were extracted based on the following NACE codes: small grocers (47.11), butchers (47.22), fishmongers (47.23), bakeries (47.24), and restaurants (56.1). Fast-food outlets and sit in restaurant were not distinguished under a specific NACE code. Supermarkets were identified using data from the Atlas of Luxembourg, and the Spatial Development Observatory, in addition to a manual search based on the websites of the supermarket brands using a web-archive search, and cross-checking the information with google street view images. In Luxembourg, convenience stores (small retail outlets that have long opening hours and mainly sell ready-to-eat food, snacks, beverages, and tobacco) are located near gas stations. To classify them, we initially identified gas stations from the Luxembourg business directory. Using the petrol company websites and Google Maps, we then verified the presence of a convenience store attached to the gas stations. We cross-verified the presence and location of all the food outlets for the using Google Maps and Google Street View, as well as the official publisher of the Luxembourg Yellow Pages (Editus.lu).

**References:**

1. European Commission. NACE Rev. 2 – Statistical classification of economic activities in the European Community. Luxembourg: Office for Official Publications of the European Communities. 2008.

# **Supplementary Table 5. Descriptive statistics of exposure to fast-food and sit-down restaurants of the study population, n=464 adults from ORISCAV-LUX 2 study**

| **Buffer sizes** | **Number** |  | **Shortest distance (m)** |  | **Spatial access** | | |
| --- | --- | --- | --- | --- | --- | --- | --- |
|  | median (IQR) |  | median (IQR) |  | median (IQR) | Q33.3 | Q66.6 |
| **Fast-food restaurants** | | | | | | | |
| 800 m | 0 (1) |  | 1458.1 (2292.0) |  | 0 (0.002) | 0 | 0 |
| 1000 m | 0 (2) |  |  |  | 0 (0.003) | 0 | 0.001 |
| **Sit-down restaurants** | | | | | | | |
| 800 m | 3 (5.5) |  | 515.1 (567.8) |  | 0.004 (0.013) | 0.002 | 0.008 |
| 1000 m | 4 (8) |  |  |  | 0.006 (0.013) | 0.003 | 0.010 |
| **Fast-food & sit-down restaurants** | | | | | | | |
| 800 m | 3 (7) |  | 495.3 (559.9) |  | 0.005 (0.015) | 0.002 | 0.009 |
| 1000 m | 4 (10) |  |  |  | 0.007 (0.017) | 0.003 | 0.012 |

# **Supplementary Table 6. Descriptive statistics of exposure to fast-food and sit-down restaurants of the study population by level of urbanicity, n=464 adults from ORISCAV-LUX II study**

| **Buffer sizes** | **Number**  median (IQR) | **Shortest distance (m)** median (IQR) | **Spatial access** median (IQR) |
| --- | --- | --- | --- |
| **Dense cities (n=109)** |  |  |  |
| **Sit-down restaurants** |  |  |  |
| 800 m | 8 (16) | 346.5 (380.1) | 0.013 (0.032) |
| 1000 m | 11 (27) |  | 0.016 (0.037) |
| **Fast-food & sit-down restaurants** |  |  |  |
| 800 m | 9 (23) | 295.0 (349.5) | 0.016 (0.039) |
| 1000 m | 14 (35) |  | 0.022 (0.047) |
| **First ring suburbs (n=88)** |  |  |  |
| **Sit-down restaurants** |  |  |  |
| 800 m | 3 (3.5) | 576.3 (525.2) | 0.004 (0.008) |
| 1000 m | 4 (5) |  | 0.005 (0.009) |
| **Fast-food & sit-down restaurants** |  |  |  |
| 800 m | 3 (3.5) | 576.3 (525.2) | 0.004 (0.008) |
| 1000 m | 4 (5.5) |  | 0.005 (0.009) |
| **Second ring suburbs (n=121)** |  |  |  |
| **Sit-down restaurants** |  |  |  |
| 800 m | 1 (3) | 663.7 (639.0) | 0.002 (0.006) |
| 1000 m | 1 (2) |  | 0.002 (0.006) |
| **Fast-food & sit-down restaurants** |  |  |  |
| 800 m | 1 (3) | 663.7 (573.7) | 0.002 (0.006) |
| 1000 m | 2 (2) |  | 0.002 (0.006) |
| **Distant suburbs (n=66)** |  |  |  |
| **Sit-down restaurants** |  |  |  |
| 800 m | 1 (3) | 748.9 (1760.0) | 0.001 (0.005) |
| 1000 m | 2 (4) |  | 0.003 (0.006) |
| **Fast-food & sit-down restaurants** |  |  |  |
| 800 m | 1 (5) | 748.9 (1760.0) | 0.002 (0.006) |
| 1000 m | 2 (5) |  | 0.003 (0.007) |
| **Former mining area (n=80)** |  |  |  |
| **Sit-down restaurants** |  |  |  |
| 800 m | 5 (5) | 423.2 (378.2) | 0.007 (0.014) |
| 1000 m | 7 (8) |  | 0.009 (0.015) |
| **Fast-food & sit-down restaurants** |  |  |  |
| 800 m | 6 (7) | 423.2 (372.8) | 0.008 (0.019) |
| 1000 m | 9 (13) |  | 0.012 (0.023) |

| **Supplementary Table 7. Estimates (β) and 95% confidence intervals (CI) for associations of spatial access to fast-food and sit-down restaurants and 24-h urinary NA excretion (mg/d)** | | | | | |  |
| --- | --- | --- | --- | --- | --- | --- |
|  | **800 m** | |  | **1000 m** | | |
|  | **β (95 CI)** | **p value** |  | **β (95 CI)** | **p value** | |
| **Spatial access to fast-food and sit-down restaurants**^a^ |  |  |  |  |  | |
| Low | ref. | - |  | ref. | - | |
| Intermediate | 133.2 (-22.5, 288.8) | 0.094 |  | 75.3 (-78, 228.6) | 0.336 | |
| High | 109.8 (-78.4, 298.1) | 0.253 |  | 105.6 (-96.1, 307.4) | 0.305 | |
| **Covariates** |  |  |  |  |  | |
| **Age** | 2.1 (-7.3, 11.4) | 0.664 |  | 2.8 (-6.3, 11.8) | 0.547 | |
| **Sex** |  |  |  |  |  | |
| Men | ref. | - |  | ref. | - | |
| Women | 461.5 (-100.6, 1023.7) | 0.107 |  | 483 (-66.8, 1032.7) | 0.085 | |
| **Resource perception** |  |  |  |  |  | |
| Easy | ref. | - |  | ref. | - | |
| Difficult | 103.3 (-91.3, 297.9) | 0.297 |  | 126.1 (-62.4, 314.6) | 0.189 | |
| Refuse to answer | 142.7 (-39.2, 324.6) | 0.124 |  | 153.5 (-38, 345.1) | 0.115 | |
| **Working status** |  |  |  |  |  | |
| Employed | ref. | - |  | ref. | - | |
| Not employed/Stay-at-home parent | -8.6 (-246.6, 229.3) | 0.943 |  | 4.6 (-212.5, 221.8) | 0.967 | |
| Disabled or retired | -41.4 (-247.6, 164.8) | 0.693 |  | -54.1 (-253, 144.8) | 0.594 | |
| **Education level** |  |  |  |  |  | |
| No diploma | ref. | - |  | ref. | - | |
| High school or vocational diploma | 12.4 (-201.6, 226.4) | 0.909 |  | 8.9 (-204.6, 222.5) | 0.934 | |
| Higher diploma | -248.8 (-476.8, -20.7) | 0.033 |  | -253.3 (-464.4, -42.2) | 0.019 | |
| **Marital status** |  |  |  |  |  | |
| Married/living with partner | ref. | - |  | ref. | - | |
| Single/never married | -202.1 (-485.8, 81.7) | 0.161 |  | -200.1 (-472.3, 72) | 0.148 | |
| Divorced/widowed | 30.2 (-146.7, 207.1) | 0.737 |  | 24.9 (-153.3, 203.2) | 0.783 | |
| **Country of birth** |  |  |  |  |  | |
| Luxembourg | ref. | - |  | ref. | - | |
| European country | 84.1 (-50, 218.3) | 0.219 |  | 89.1 (-47.3, 225.5) | 0.2 | |
| Not European country | 100.6 (-213.3, 414.5) | 0.528 |  | 72.6 (-236.1, 381.4) | 0.643 | |
| **Presence of a child in the household** |  |  |  |  |  | |
| No | ref. | - |  | ref. | - | |
| Yes | -67 (-239.7, 105.7) | 0.446 |  | -61.5 (-227.2, 104.2) | 0.466 | |
| **Great importance attached to eating balanced meal for good health** |  |  |  |  |  | |
| Yes | ref. | - |  | ref. | - | |
| No | 2.1 (-141.3, 145.5) | 0.977 |  | 9.2 (-134.6, 153) | 0.9 | |
| **Great importance attached to maintaining normal weight for good health** |  |  |  |  |  | |
| Yes | ref. | - |  | ref. | - | |
| No | 132.9 (-15.9, 281.7) | 0.080 |  | 122.7 (-27.8, 273.1) | 0.11 | |
| **Retail Food Environment Index (mRFEI)**^a^ |  |  |  |  |  | |
| Low | ref. | - |  | ref. | - | |
| Intermediate | 47.9 (-182.7, 278.5) | 0.683 |  | 33.3 (-174.4, 241) | 0.753 | |
| High | 76.7 (-68.4, 221.8) | 0.300 |  | 30.6 (-126.6, 187.9) | 0.701 | |
| **Neighborhood SES** |  |  |  |  |  | |
| Factor 1 | -25.6 (-102.8, 51.7) | 0.516 |  | -17.1 (-94.4, 60.2) | 0.664 | |
| Factor 2 | -12.5 (-81.3, 56.2) | <.0001 |  | -5.5 (-74, 62.9) | <.0001 | |
| **Interaction terms** |  |  |  |  |  | |
| **Spatial access to restaurants**^a^***Great importance attached to eating balanced meal for good health** |  |  |  |  |  | |
| Low*Yes | ref. | - |  | ref. | - | |
| Intermediate*Yes | -91.3 (-309.8, 127.2) | 0.412 |  | -117 (-325.7, 91.7) | 0.272 | |
| High*Yes | -39.8 (-288.1, 208.5) | 0.753 |  | -59 (-317.3, 199.3) | 0.654 | |
| Low*No | ref. | - |  | ref. | - | |
| Intermediate*No | 259.4 (19.5, 499.4) | 0.034 |  | 270.3 (20.8, 519.8) | 0.034 | |
| High*No | 357.6 (135.6, 579.7) | 0.002 |  | 267.6 (47.2, 488.0) | 0.018 | |
| **Age*Sexe** |  |  |  |  |  | |
| AGE*men | ref. | - |  | ref. | - | |
| AGE*women | -31.8 (-42.1, -21.5) | <.0001 |  | -31.8 (-42.1, -21.5) (<.0001, ) | <.0001 | |

^a^ categorization into low, intermediate or high based on tertiles of the observed distribution of the corresponding variable.

# **Supplementary Table 8. Estimates (β) and 95% confidence intervals (CI) for associations of spatial access to fast-food and sit-down restaurants and 24-h urinary Na excretion (mg/d), by different road network buffer sizes in the non-imputed dataset**

|  | **800 m** | |  | **1000 m** | |  |
| --- | --- | --- | --- | --- | --- | --- |
| **Tertiles of spatial access** | **β (95% CI)** | **P-value** |  | **β (95% CI)** | **P-value** |  |
| **Model 1**^a^ |  |  |  |  |  |  |
| Low | ref. | - |  | ref. | - |  |
| Intermediate | 110.5 (-32.1, 253.1) | 0.128 |  | 63.8 (-78.4, 206) | 0.378 |  |
| High | 136.8 (-8.2, 281.9) | 0.064 |  | 141.1 (-4.1, 286.2) | 0.057 |  |
| **Model 2^b^** |  |  |  |  |  |  |
| Low | ref. | - |  | ref. | - |  |
| Intermediate | 110.7 (-35.9, 257.3) | 0.139 |  | 76.3 (-74.2, 226.8) | 0.320 |  |
| High | 159.5 (-19, 338) | 0.080 |  | 185.2 (-6.2, 376.6) | 0.058 |  |

^a^ Model 1 is adjusted for sex, age, country of birth (Luxembourg, European country or non-European country), resource perception (difficult, easy or refuse to answer), educational level (no diploma, secondary education or higher diploma), working status (employed, not employed, stay-at-home parent or disabled/ retired), marital status (married/living with partner, single/never married or divorced/widowed), presence of a child in the household (yes or no), great importance attached to eating balanced meal for good health (yes or no), great importance attached to maintaining normal weight for good health (yes or no).

^b^ Model 2 = Model 1 + tertiles of modified Retail Food Environment Index (mRFEI), as well as two scores of neighborhood SES derived from PCA.

# **Supplementary Table 9. Estimates (β) and 95% confidence intervals (CI) for associations of spatial access to sit-down restaurants and 24-h urinary Na excretion (mg/d), by different road network buffer sizes**

|  | **800 m** | |  | **1000 m** | |  |
| --- | --- | --- | --- | --- | --- | --- |
| **Tertiles of spatial access** | **β (95% CI)** | **P-value** |  | **β (95% CI)** | **P-value** |  |
| **Model 1**^a^ |  |  |  |  |  |  |
| Low | ref. | - |  | ref. | - |  |
| Intermediate | 127.5 (-21.5, 276.4) | 0.094 |  | 82.4 (-62.2, 227) | 0.264 |  |
| High | 112.8 (-32.8, 258.4) | 0.129 |  | 116.3 (-33.2, 265.7) | 0.127 |  |
| **Model 2**^b^ |  |  |  |  |  |  |
| Low | ref. | - |  | ref. | - |  |
| Intermediate | 121.4 (-30.1, 272.9) | 0.116 |  | 80.9 (-69.2, 230.9) | 0.291 |  |
| High | 117.5 (-64.2, 299.3) | 0.205 |  | 121.3 (-73.6, 316.2) | 0.222 |  |

^a^ Model 1 is adjusted for sex, age, country of birth (Luxembourg, European country or non-European country), resource perception (difficult, easy or refuse to answer), educational level (no diploma, secondary education or higher diploma), working status (employed, not employed, stay-at-home parent or disabled/ retired), marital status (married/living with partner, single/never married or divorced/widowed), presence of a child in the household (yes or no), great importance attached to eating balanced meal for good health (yes or no), great importance attached to maintaining normal weight for good health (yes or no).

^b^ Model 2 = Model 1 + tertiles of modified Retail Food Environment Index (mRFEI), as well as two scores of neighborhood SES derived from PCA.

# **Supplementary Table 10. Estimates (β) and 95% confidence intervals (CI) for associations of counts of fast-food, sit-down restaurants, and fast-food and sit-down restaurants and 24-h urinary Na excretion (mg/d), by different road network buffer sizes in the non-imputed dataset**

|  | **800 m** | |  | **1000 m** | |  |
| --- | --- | --- | --- | --- | --- | --- |
|  | **β (95% CI)** | **P-value** |  | **β (95% CI)** | **P-value** |  |
| **Presence of fast-food restaurants** | | | | | |  |
| **Model 1**^a^ |  |  |  |  |  |  |
| Absence | ref. | - |  | ref. | - |  |
| Presence | -89.4 (-219.0, 40.2) | 0.176 |  | -33.0 (-154.8, 88.8) | 0.595 |  |
| **Model 2^b^** |  |  |  |  |  |  |
| Absence | ref. | - |  | ref. | - |  |
| Presence | **-175.7 (-340.3, -11.2)** | **0.036** |  | -113.3 (-274.6, 48.0) | 0.168 |  |
| **Count of sit-down restaurants** | | | | | |  |
| **Model 1**^a^ |  |  |  |  |  |  |
| Low | ref. | - |  | ref. | - |  |
| Intermediate | 45.0 (-104.5, 194.4) | 0.555 |  | 23.8 (-131.0, 179.5) | 0.764 |  |
| High | 53.0 (-92.0, 198.1) | 0.474 |  | 29.5 (-130.1, 189.2) | 0.716 |  |
| **Model 2^b^** |  |  |  |  |  |  |
| Low | ref. | - |  | ref. | - |  |
| Intermediate | 29.5 (-125.5, 184.4) | 0.709 |  | -0.4 (-161.9, 161.1) | 0.996 |  |
| High | 33.3 (-168.4, 235.1) | 0.746 |  | -45.2 (-278.6, 188.2) | 0.704 |  |
| **Count of fast-food and sit-down restaurants** | | | | | |  |
| **Model 1**^a^ |  |  |  |  |  |  |
| Low | ref. | - |  | ref. | - |  |
| Intermediate | 17.9 (-124.5, 160.4) | 0.805 |  | 23.2.8 (-132.7, 179.1) | 0.771 |  |
| High | 51.98 (-102.3, 206.3) | 0.509 |  | 38.1 (-121.6, 197.8) | 0.640 |  |
| **Model 2^b^** |  |  |  |  |  |  |
| Low | ref. | - |  | ref. | - |  |
| Intermediate | 3.5 (-145.2, 152.2) | 0.963 |  | 1.3 (-161.4, 164.1) | 0.987 |  |
| High | 44.7 (-183.6, 273.0) | 0.701 |  | -26.6 (-268.3, 215.2) | 0.829 |  |

^a^ Model 1 is adjusted for sex, age, country of birth (Luxembourg, European country or non-European country), resource perception (difficult, easy or refuse to answer), educational level (no diploma, secondary education or higher diploma), working status (employed, not employed, stay-at-home parent or disabled/ retired), marital status (married/living with partner, single/never married or divorced/widowed), presence of a child in the household (yes or no), great importance attached to eating balanced meal for good health (yes or no), great importance attached to maintaining normal weight for good health (yes or no).

^b^ Model 2 = Model 1 + tertiles of modified Retail Food Environment Index (mRFEI), as well as two scores of neighborhood SES derived from PCA.

**
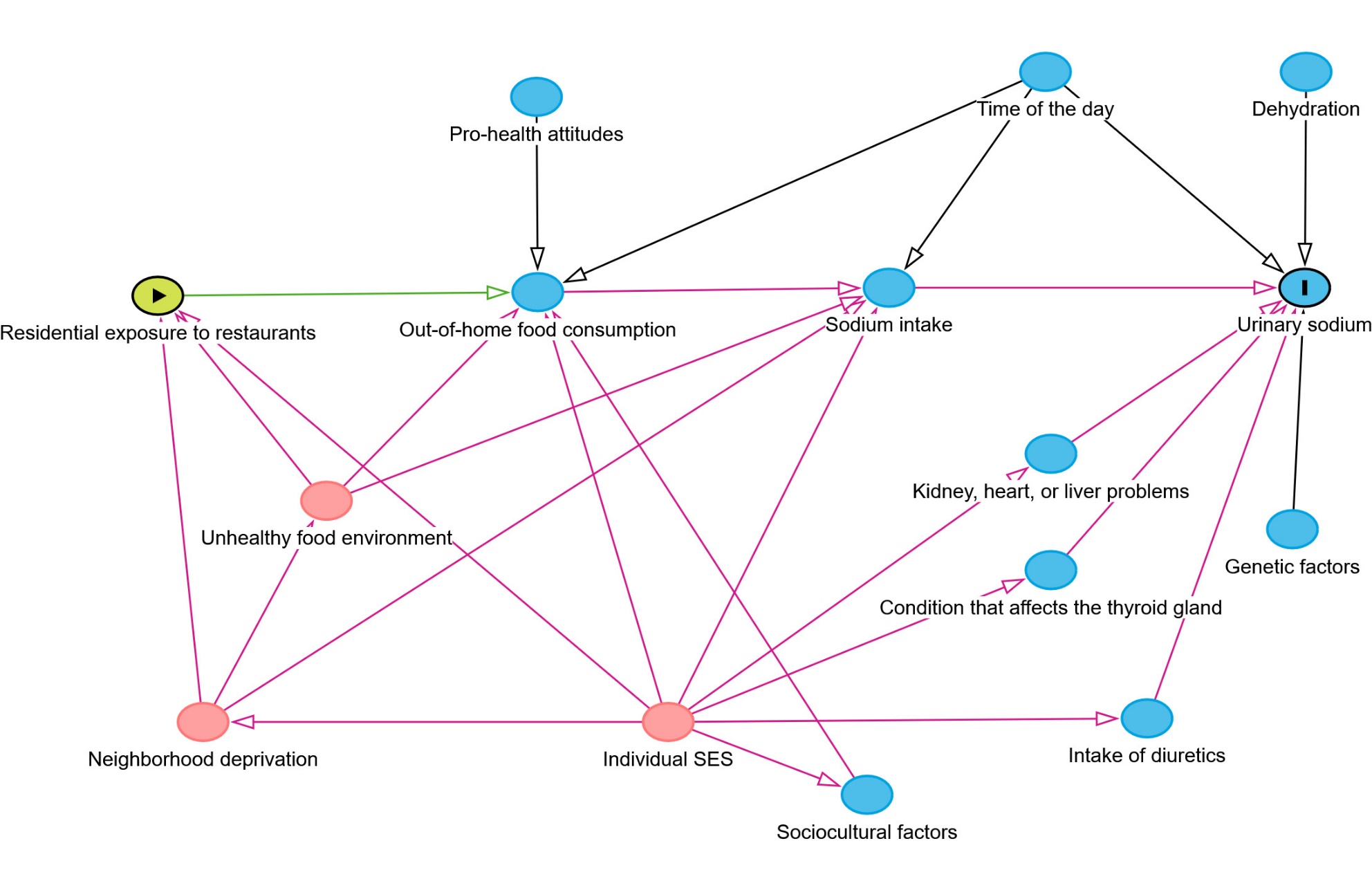
**

# **Supplementary Figure 1. DAG Directed Acyclic Graph (DAG) representing the causal pathways between exposure to fast-food and sit-down restaurants, and 24-hour urinary Na excretion**

Created on http://dagitty.net. The outcome variable is identified as a blue oval with the letter "I"; the exposure variable is identified as green ovals. Variables on the causal pathways are identified as blue ovals, and potential confounders as red ovals.


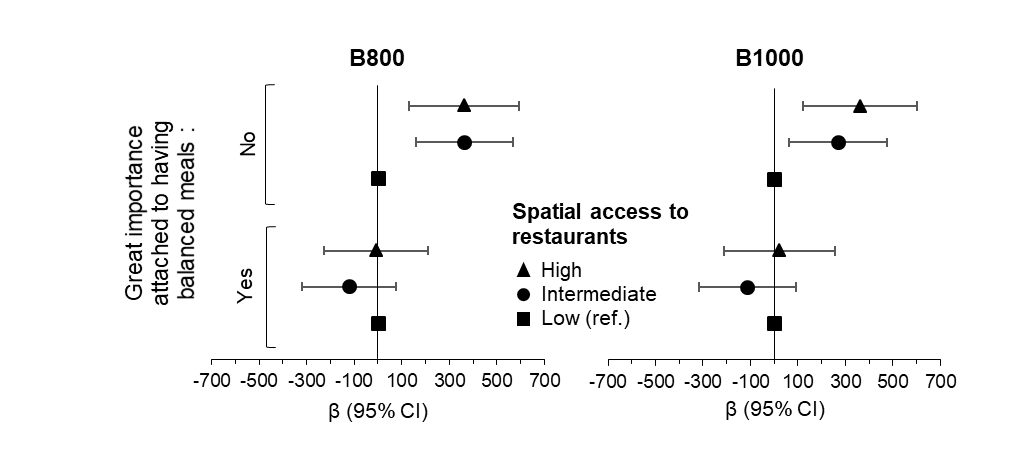


# **Supplementary Figure 2. Estimates (β) and 95% confidence intervals (CI) for associations of spatial access to restaurants and 24-h urinary Na excretion (mg/d), at 800 and 1000 m, according to health-conscious eating habits in the non-imputed dataset.**

Fully adjusted model (Model 2)


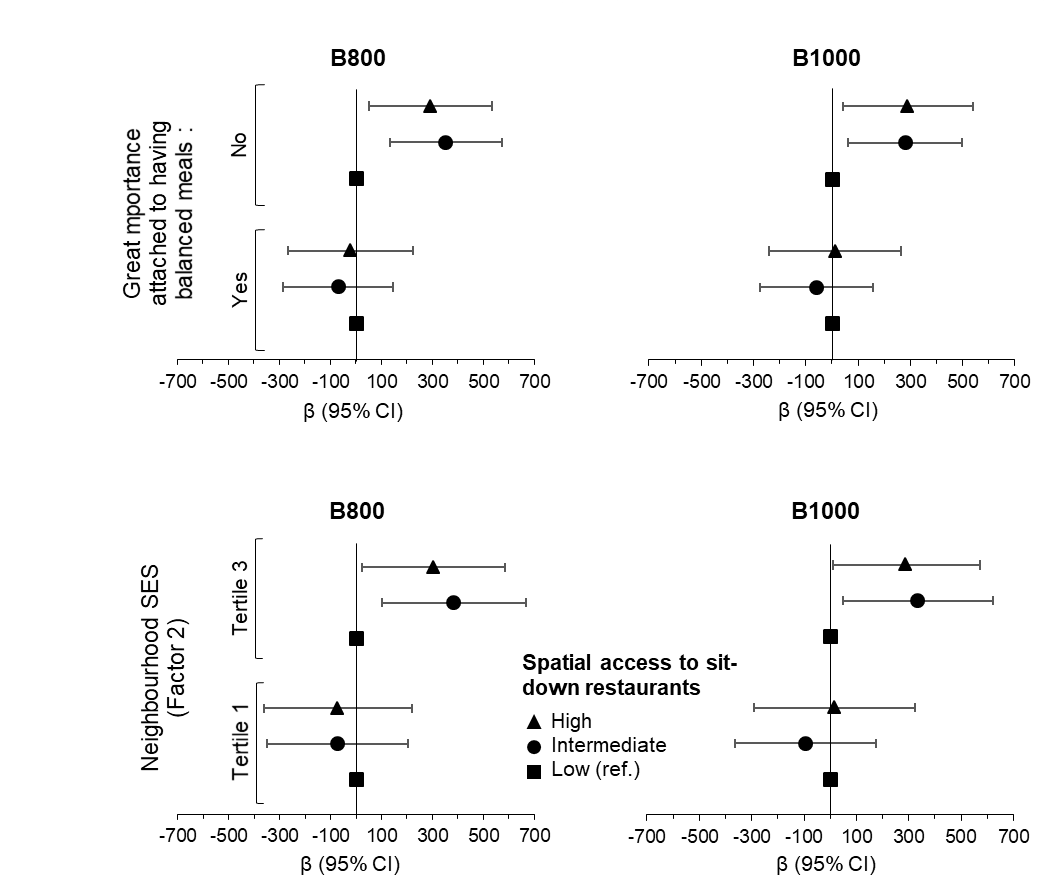


# **Supplementary Figure 3. Estimates (β) and 95% confidence intervals (CI) for associations of spatial access to sit-down restaurants and 24-h urinary Na excretion (mg/d), at 800 and 1000 m, according to health-conscious eating habits and neighborhood SES (Factor 2).**

Fully adjusted model (Model 2)
